# Fracture Strength and Stress Distribution in Premolars Restored with Cast Post-and-Cores or Glass-Fiber Posts Considering the Influence of Ferule

**DOI:** 10.1155/2019/2196519

**Published:** 2019-01-03

**Authors:** Atais Bacchi, Ricardo Armini Caldas, Daniel Schmidt, Mauricio Detoni, Matheus Albino Souza, Doglas Cecchin, Ana Paula Farina

**Affiliations:** ^1^Graduate Program in Dentistry, Meridional Faculty, IMED, Passo Fundo, RS, Brazil; ^2^Department of Prosthodontics and Periodontology, Piracicaba Dental School, State University of Campinas, FOP/UNICAMP, Piracicaba, SP, Brazil; ^3^Graduate Program in Dentistry, University of Passo Fundo, Passo Fundo, RS, Brazil

## Abstract

**Objectives:**

The aim of this study was to evaluate the influence of ferule and the post type on the fracture strength and stress distribution in premolars.

**Materials and Methods:**

Forty human mandibular premolars were decoronated, allocated in four groups, and restored as follows: CPC-NF: cast post-and-core and absence of ferule; CPC-F: cast post-and-core and presence of ferule; FPC-NF: glass-fiber posts and absence of ferule; FPC-F: glass-fiber posts and presence of ferule. The fracture strength (FS) and failure patterns were evaluated. Finite element analysis (FEA) evaluated the stress distribution.

**Results:**

FS did not differ between CPCs and FPC either in presence or in absence of ferule. The presence of ferule increased FS with both post types. Mean values of FS for ferule groups were higher than functional or parafunctional loads reported in literature, which was not the case for FPC-NF when compared to parafunctional loads. FEA with a functional load showed slightly higher compressive stresses in dentin in the group CPC-NF, which was much lower than the compressive strength of dentin. Lower percentage of catastrophic failures was observed in nonferule groups irrespective of post type, which was explained by the stress concentration in the cervical root region when FEA with the FS load was simulated.

**Conclusion:**

Ferule effect was shown to be more important than post type in the analysis. Both posts showed potential to withstand functional loads irrespective of presence of ferule. However, the mean FS was lower than parafunctional loads for FPC in the absence of ferule.

## 1. Introduction

Teeth with severe loss of coronal structure constitute a daily challenge in clinical practice. In general, when the remaining tooth structure is not considered adequate to retain a restoration, intracanal posts are used. The literature suggests that at least 1.5 mm of coronal structure would improve the biomechanical behavior of the restoration even with the use of intracanal posts [1]. After encircled by the crown, this tooth structure provides the so-called “ferule effect” [1].

Besides the interest in keeping the remaining coronal structure, the intracanal post material is still a topic of discussion in literature. For several years, the metallic cast post-and-cores (CPCs) were the most utilized, assuming that their higher elastic modulus would ensure better support to the restoration. Moreover, the adequate juxtaposition to dentin of individualized CPCs is considered responsible for the conjunct withstand higher loads when compared to prefabricated posts [2, 3]. Besides the fact that these concepts were accepted for years, recent studies are in disagreement to the superiority of fracture strength caused by CPCs [4–6].

The increase in esthetic needs and the appeal to develop intracanal posts with elastic modulus closer to that of dentin led to the development of glass-fiber posts. It is also worth mentioning that prefabricated posts can provide a reduced treatment time as the post could be bonded at the day of the canal preparation. Differences in the fracture strength between CPCs and glass-fiber posts might depend on other factors than simple post material. These factors in general involve tooth region, load incidence and direction, post diameter, and amount of remaining coronal structure [1, 3]. Therefore, it is difficult to establish a definitive consensus about the comparison of fracture strength between teeth restored with these different post materials.

The literature is still controversial about what would be the best post option either in the presence or in the absence of remaining coronal structure (and consequently ferule effect). Therefore, the aim of this study was to evaluate the fracture strength and stress distribution in mandibular premolars restored with cast post-and-cores or glass-fiber posts considering the influence of the ferule effect. The null hypothesis was that the post type and ferule effect would not influence the biomechanical behavior of the mandibular premolars.

## 2. Materials and Methods

### 2.1. Laboratory Analysis

Forty single-rooted human premolars (n=10) free of caries or cracks were selected for the study. Teeth were stored in thymol 0.9% during the experiment.

The specimens were randomly allocated to the studied groups:

CPC-NF: cast post-and-core in teeth without remaining coronal structure;

CPC-F: cast post-and-core in teeth with remaining coronal structure;

FPC-NF: glass-fiber post (Number 3, Reforpost glass-fiber; Angelus, Londrina, PR, Brazil) relined with composite in teeth without remaining coronal structure;

FPC-F: glass-fiber post (Number 3, Reforpost glass-fiber; Angelus, Londrina, PR, Brazil) relined with composite in teeth with remaining coronal structure.

Teeth of NF groups were decoronated at 12 mm from the apex and the F groups at 14 mm, with a diamond disc (KG Sorensen, Barueri, SP, Brazil). The endodontic treatment was performed. Each root canal was prepared at 1 mm of the radiographic apex and instrumented up to a file size #35 (Dentsply-Maillefer, Ballaiguess, Switzerland) with a conventional step-back technique. Canals were irrigated with 1% sodium hypochlorite for detersion. The canals were filled with an ISO 35 primary gutta-percha master cones (Tamari; Tamariman Industrial Ltd, Macaçaruru, AM, Brazil), accessories gutta-percha cones (Tanari; Tanariman Industrial Ltda.), and eugenol-free sealer (Sealer 26, Dentsply Ind. e Com. Ltda., Petrópolis, RJ, Brazil) [5]. The root canal was prepared up to 8 mm for teeth in NF groups and 10 mm for those in F groups, with Largo reamers (Dentsply Maileffer) up to number #3 (Ø 1.1 mm). A two-millimeter tooth preparation of the cervical portion was performed for the F groups with diamond bur (#3216, KG Sorensen, Barueri, São Paulo, SP, Brazil) in a high-speed hand-piece to create the so-called “ferule effect.”

Cast post-and-cores were confectioned using matrices made of low-shrinkage polymethyl methacrylate (Duralay, Reliance Dental Mfg. Co., Worth, Illinois, USA) and prefabricated pins (Pin Jet, Angelus). The structures were casted in copper-aluminum alloy (Durabond, São Paulo, SP, Brazil) by the lost-wax technique. An acetate matrix was used to standardize the core dimensions. The cores had a final height of 6 mm.

For FPC groups, the fiber post surface was cleaned with ethanol and treated with silane (Silano, Angelus) during 60 s and received a layer of hydrophobic adhesive (Adper Scothbond, 3M ESPE), which was air-dried for solvent elimination and light activated for 20 s at 1,200 mW/cm^2^ (Radii-Cal, SDI, Bayswater, Australia). The fiber post was relined with composite resin (Filtek Z350; 3M ESPE). All posts of the study were luted to root canal with self-adhesive resin cement (RelyX U200; 3M ESPE) according to manufacturer's instruction. The tooth remaining coronal structure of FPC groups received the application of an etch-and-rinse adhesive (Adper Scotchbond, 3M ESPE), and composite cores (Z350; 3M ESPE) were built with the aid of an acetate matrix.

Matrices of copings traditionally used for metal-ceramic restorations were made of polymethyl methacrylate (Duralay, Reliance Dental Mfg. Co.) and casted in nickel-chromium alloy (Durabond, São Paulo, SP, Brazil) by the lost-wax technique. The copings were bonded to the cores with self-adhesive resin cement (RelyX U200; 3M ESPE). Specimens were included in a polystyrene resin (Cristal; Piracicaba, São Paulo, Brazil) with simulation of the periodontal ligament using elastomer (Impregum Soft, 3M ESPE). Before immersion in resin, the root was evolved with a 0.6 mm thick foil (Adapta foil; BEGO, Bremen, Germany). The tooth was positioned into the cylinder and the resin was applied. After the first signals of resin polymerization, the tooth was removed along its long axis. The foil was removed and elastomeric material (Impregum Soft, 3M ESPE, St. Paul, MN, USA) was injected into the polystyrene resin blocks and the tooth was repositioned, creating a standardized layer that simulates the periodontal ligament (approximately 60 *μ*m) [5].

Specimens were aged with 250,000 mechanical cycles, which has been considered to mimic one year of clinical function [4, 7]. A standard 100 N load and frequency of 4 Hz were also adopted [5, 6]. The ageing regimen aimed to mimic debonding, dentine, or composite microcracking caused during function. Fracture strength (FS) was tested in a universal testing machine (Instron 1144, Instron Corporation, Canton, MA, USA). The load was applied at a 135° angle to the root long axis with a crosshead speed of 0.5mm/min. The failure pattern analysis, developed with an X4 binocular loupe (Bio-Art Equipamentos Odontologicos Ltda, São Carlos, SP, Brazil), was performed to define the failure patterns: Type I, fracture in the crown-core bonding interface; Type II, fracture in the root cervical third; Type III, fracture in the root middle third; Type IV, fracture in the root apical third; Type V, vertical fracture.

### 2.2. Finite Element Analysis

One tooth with the standard dimensions used in the study was scanned via computed tomography (*μ*CT) (SkyScan 1174v2; Bruker microCT, Kontich, Belgium). Tridimensional numerical models were created with computer-aided design software (Rhinoceros 3D, Seattle, WA, USA). The simulated periodontal ligament (polyether) and cancellous bone (polystyrene resin) were included. Four finite element models were created, representing the experimental groups.

The models were exported to finite element analysis software (ANSYS Workbench 11, Ansys Inc., Pittsburg, PA, USA). The shape of the element was tetrahedral with 10 nodes and 0.8 mm length, with 3 rotational degrees of freedom and 3 translational degrees of freedom at each node. The stability of the model was checked, the mesh was refined in the regions of interest, and convergence tests with 6% were made until the mesh did not influence the results. The mesh generation resulted in about 700,000 elements and 1,000,000 nodes per model. The material properties were obtained from published data as present in Table 1.

Two load conditions were simulated. First, groups were compared after application of a mean bite force of 300 N [8]. Second, each group was loaded with the respective fracture strength obtained in the laboratory analysis. The load was applied under the lingual cingulum at 135° to the long axis of the tooth. Model movements were restricted in all directions at the base nodes of the polystyrene resin base.

All tooth structures and materials were considered elastic, isotropic, linear, and homogeneous, except the orthotropic glass-fiber posts (Table 1). For the evaluation with the load of the fracture strength, failure at post/dentin interface was considered in glass-fiber groups before tooth fracture [9]. For that, an attrition coefficient of 0.3 was applied.

Maximum principal stress (*σ*_max_, tensile) and minimum principal stress (*σ*_min_, compressive) in dentin were obtained and evaluated quantitatively (numerically) and qualitatively (stress location).

### 2.3. Statistical Analysis

Data of fracture strength was evaluated by two-way ANOVA followed by Tukey's test (95%).

## 3. Results

### 3.1. Laboratory Analysis

The groups CPC-F and FPC-F were similar to each other (*p*>0.05) and presented higher fracture strength than samples with the same intracanal post without ferule (NF) (*p*<0.001). Differences between CPC-NF and FPC-NF were not significant (*p*>0.05). Results are presented in Table 1.

**Figure 1:**
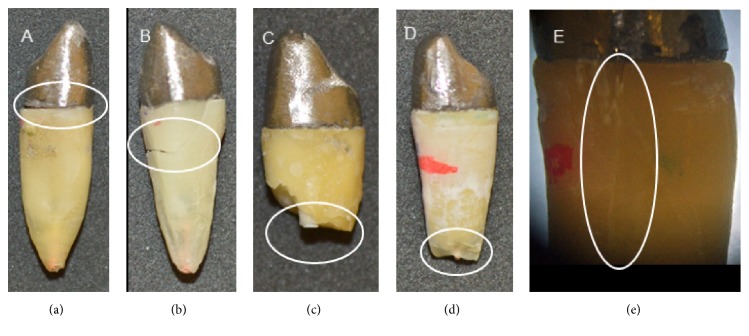
Examples of failure patterns observed in the study. (a) Type 1, cementation fracture. (b) Type 2, fracture in the cervical third. (c) Type 3, fracture in the middle third. (d) Type 4, fracture in the apical third. (e) Type 5, longitudinal fracture. (a) and (b) repairable fractures; (c), (d), and (e) irreparable fractures.

The most favorable failure pattern was seem in the group restored with FPC-NF (70%) followed by CPC-NF (60%). The group CPC-F showed the greater amount of irreparable fractures (70%). An equivalent distribution of repairable and irreparable fractures was observed in the FPC-F group. Failure patterns for each group can be observed in Table 2 and representative imagens are shown in Figure 1.

### 3.2. Finite Element Analysis

**Figure 2:**
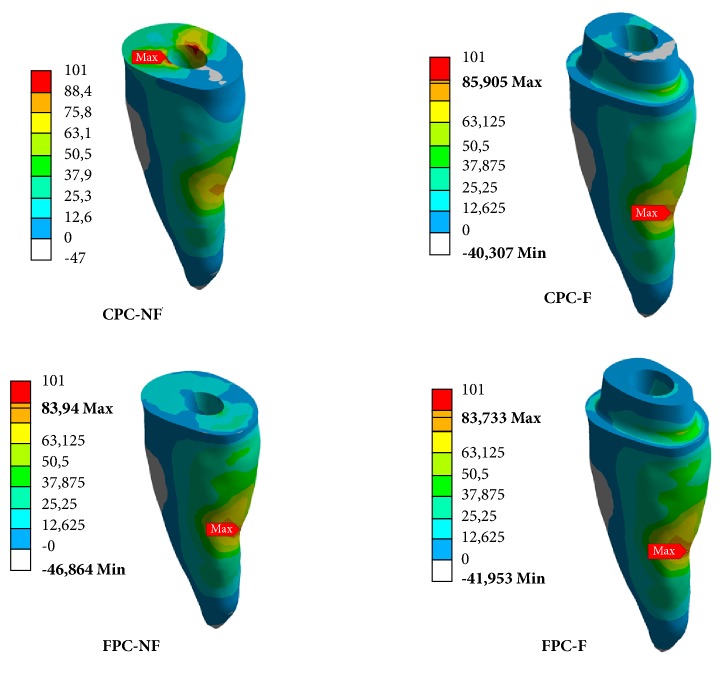
Representation of the *σ*_max_ and *σ*_min_ stress concentration in the groups with a standard load of 300 N.

In the evaluation with a mean load of 300 N (Table 3, Figure 2), the group CPC-NF showed the highest *σ*_max_ and *σ*_min_ in dentin. The other groups did not present relevant differences among them. The local of *σ*_max_ stress concentration for CPC-NF was the root canal entry (cervical third), while for other groups the region was the medium root third.

**Figure 3:**
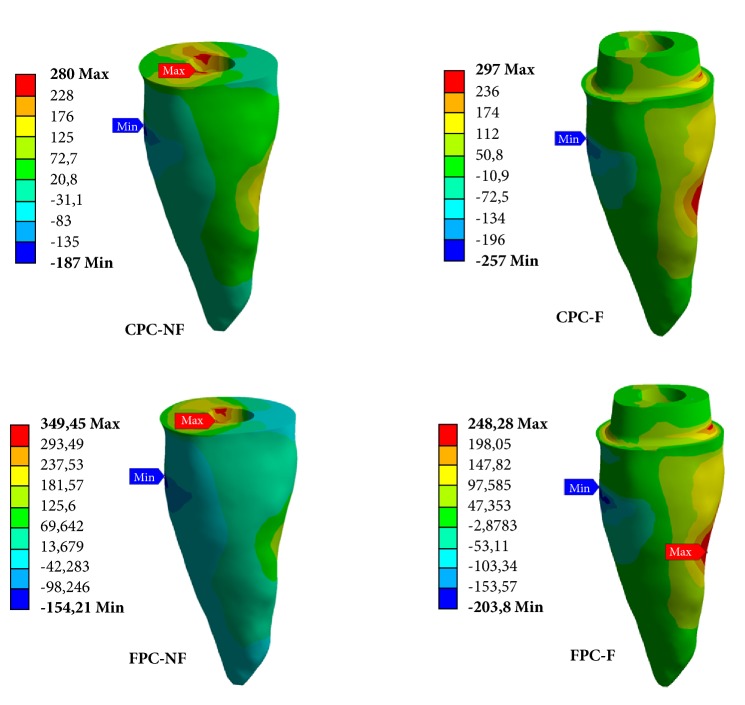
Representation of the *σ*_max_ and *σ*_min_ stress concentration in the groups when the load of fracture strength teste was applied.

When each group was loaded with the respective fracture strength obtained in the laboratory analysis (Table 4 and Figure 3), the group FPC-NF led to relevant higher *σ*_max_ stress concentration in dentin. The lowest *σ*_max_ and *σ*_min_ among groups were presented by FPC-F. Groups restored with CPC did not present relevant differences in *σ*_max_ between them. The absence of ferule led to relevant higher *σ*_min_ for groups of both post systems.

The qualitative analysis (Figure 3) showed that groups without ferule present *σ*_max_ at cervical portion of the root canal. *σ*_min_ was located between cervical and middle root thirds in all groups. Groups containing ferule had stress concentration in the ferule region but the highest stress was transferred to the medium root third.

## 4. Discussion

This study showed that the “ferule effect” influences the biomechanical behavior of endodontically treated mandibular premolars. However, influence of post type was not observed. Therefore, the null hypothesis was partially accepted. The ferule effect provided an increase in fracture strength, irrespective of the post type used. Ferule effect is characterized by the presence of parallel remaining dentin walls that produce a protective behavior after encircled by the crown, reducing the stresses in the core structure, and better distributing stress along the remaining tooth tissue [4, 6, 10]. This effect might be also responsible by the similarity of stress distribution in the simulated models with ferule in FEA.

The fracture strength of teeth restored with CPC or FPC in literature is controversial. However, this study agreed with previous reports where both post types were similar in fracture strength either in the presence [6] or in absence of ferule [5]. CPCs have been considered to withstand higher loads because of their higher elastic modulus [7, 11]. However, it is also suggested that, during load application, the CPCs transmit more stress to the tooth structure and a slow crack growth causes a successive failure of the post-cement-root dentin interface [4, 12]. CPC will therefore act like a wedge and the energy accumulated in the inner post is transferred to dentin causing failure [4, 12]. Besides the fact that the glass-fiber posts are less resistant to failure than CPC, the elastic modulus closer to that of dentin is less likely to induce dentin crack initiation and propagation [4].

The values of fracture strength observed in this study (ranging from 620 N to 1,266 N) are considered higher than the mean bite force observed in literature, which is 304.9 N for men and 284.9 N for women [8]. This is probably why randomized controlled clinical trials (RCTs) fail to point out differences between the longevity of CPC and FPC even in the absence of remaining coronal structure and, therefore, without ferule effect [13]. In the case of patients with parafunction, a study registered a mean nocturnal bite force up to 81.2 kgf (795.7 N) [14]. Therefore, the use of fiber posts in the absence of ferule could be critical, considering the mean values of fracture strength of the present study.

Considering the adoption of mean functional loads (300 N) in FEA, no relevant differences were observed in stress distribution in the presence of remaining coronal structure, which has already been discussed as an influence of the ferule effect. In the absence of ferule, CPC led to an increase of about 10% tensile stress and 200% compressive stress distribution in dentin in comparison to FPC, which might be attributed to its higher elastic modulus [2]. Moreover, the higher stresses for CPC-NF were observed in the cervical root third, whereas for other system the stresses were better distributed along the root structure. Besides the increase in compressive stress (from 4 to 12 MPa), all values are much lower than compressive strength of dentin presented in literature (432 MPa) [15]. Therefore, it is possible to assume that the stress generated is not critical for dentin fracture. This is also supported for a meta-analysis where clinical fractures observed in teeth restored with CPC were not more predominant than that observed with FPC [16].

The failure pattern analysis revealed a greater number of repairable fractures in the groups without ferule, with a similar percentage between post types (70% for CPC and 60% for FPC). A finite element analysis was carried out with the respective FS of the groups to evaluate the stress distribution pattern. It was observed that, in the absence of ferule, the maximum principal (tensile) stresses concentrate predominantly in the cervical portion of the tooth structure, while for the ferule groups, especially CPC, in which 70% of irreparable fractures were observed, the stresses were observed in the ferule region and along the root structure. However, it is worth mentioning that neither physiologic nor parafunctional loads reported in literature are as high as the fracture strength reported in ferule groups.

An interesting result to expose the importance of the ferule effect is that even though a much higher load was applied in the ferule groups in the second finite element analysis, they presented lower compressive stresses in comparison to the respective post type evaluated without ferule. Still, the tensile stresses were similar in the CPC groups, with a much higher load applied in the ferule model. An inverse relation of load vs. tensile stress was observed for FPC groups, as the tensile stress was lower for the ferule group in which a much higher load was applied. This information can help to explain the lower mean value of fracture strength of FPC-NF in the study. These results show how ferule effect provides a better stress distribution along the remaining structure. The data of this study are relevant to show that biomechanical behavior is most dependent on the remaining coronal structure than the post system used.

A recent manuscript showed that debonding should be simulated between glass-fiber post and dentin to better associate the FEA with the laboratory data [9]. The present manuscript is the first to adopt this parameter of FEA in association with a laboratory test in the same study. This validation of methods might help to explain why the results of the present evaluation are compatible even with clinical studies that compared glass-fiber with cast post-and-cores in a controlled population [13].

## 5. Conclusions

The presence of ferule led to higher fracture strength irrespective of the post type. CPC and FPC led to similar fracture strength either in the presence or in absence of ferule.

Teeth without ferule presented the most favorable failure patterns, irrespective of post system used, which could be explained by the stress concentration at the root canal entry in the cervical third. As better stress distribution along root was observed in teeth with ferule, greater load and energy accumulation was possible, having as a consequence the most profound fractures.

When samples were subjected to a standard functional bite load in FEA, the group CPC-F presented similar qualitative and quantitative stress in dentin in comparison to FPC-F. The dentin received higher stresses in teeth without ferule when restored with CPC. However, all evaluated configurations of study showed to be adequate to withstand functional loads.

Both post systems can support parafunctional loads with the presence of ferule. Greater concern does exist for the FS of the FPC group in the absence of ferule compared to reports of parafunctional loads.

## Figures and Tables

**Table 1:** Mechanical properties of materials simulated in the study.

Material	E (GPa)	Poisson ratio (*v*)	Shear modulus (GPa)
Dentin [17]	18	0.31	
Polyether [18]	5 × 10^−2^	0.45	
Polystyrene resin [18]	13.5	0.31	
Composite resin [18]	15.8	0.24	
Stainless steel [19]	19.3	0.3	
Cu-Al alloy [12]	109	0.33	
Nickel-chromium alloy [20]	205	0.33	
Resin cement [21]	18.6	0.28	
Gutta percha [22]	1.4 × 10^−1^	0.45	
Glass Fiber post [23]	X=37	Xy=0.27	Gxy=3.1
	Y=9.5	Xz=0.34	Gxz=3.5
	Z=9.5	Yz=0.27	Gyz=3.1

**Table 2:** The mean fracture strength (N), standard deviation (±SD), and failure mode for each experimental condition.

Groups	Fracture strength (N)	Failure mode distribution						
		Type 1	Type 2	Type 3	Type 4	Type 5	Repairable	Irreparable
FPC - F	1,031.93 (383.10)^Aa^	-	5	5	-	-	5	5
FPC - NF	620.15 (241.82)^Ba^	2	5	1	1	1	7	3
CPC - F	1,266.57 (352.76)^Aa^	-	3	7	-	-	3	7
CPC - NF	809.72 (206.11)^Ba^	-	6	3	-	1	6	4

Main effect: Ferule, *p* < 0.001 Post type, *p* = 0.112								
Interaction effect: Ferule x post type, *p* = 0.080								

FPC, fiber post relined with composite resin; CPC, cast post-and-core. F – Ferule; NF – no ferule. Uppercase letters represent the significance for the factor “ferule” within the same post material; lowercase letters represent significance for the factor “post material” within the same ferule condition.

**Table 3:** Stress results (MPa) in dentin in samples with a standard load of 300 N.

Group	Load (N)	*σ*_max(+)_	*σ*_min(+)_
CPC-NF	300	101	14
CPC-F		86	4
FPC-NF		84	3
FPC-F		84	2

**Table 4:** Stress results (MPa) in dentin for the finite element analysis with the load of fracture strength of each group.

Group	Load (N)	*σ*_max(+)_	*σ*_min(+)_
CPC-NF	806	280	41
CPC-F	1272	297	11
FPC-NF	622	349	19
FPC-F	1032	248	7

## Data Availability

All the data used to support the findings of this study are available from the corresponding author upon request.
